# Factors predicting eHealth literacy: A profile of California, USA

**DOI:** 10.1016/j.pecinn.2025.100410

**Published:** 2025-06-28

**Authors:** Ronald W. Berkowsky, Brandon Almanza, Jasmine Garcia, Noah Graza

**Affiliations:** a California State University Channel Islands, 1 University Dr., Camarillo, CA 93012, United States of America; b West Coast University, United States of America; c San Diego State University, United States of America

**Keywords:** eHealth, eHealth literacy, Computer literacy, Health literacy, Information literacy, Internet, Information and communication technologies (ICTs)

## Abstract

**Objective:**

Given the known benefits of enhanced eHealth literacy in promoting patient health, identifying segments of the patient population at risk for low eHealth literacy can provide avenues for health professionals to promote enhanced eHealth skills through targeted outreach and intervention. The purpose of this study was to examine factors associated with eHealth literacy and to identify disparities in eHealth literacy in California.

**Methods:**

Using survey data from the 2020 CALSPEAKS survey, ordinary least squares regression was performed on measures of self-assessed eHealth literacy to identify associated factors (*N* = 780).

**Results:**

Findings showed that the strongest and most consistent predictors of eHealth literacy included self-rated health, a proxy measure for religiosity, Internet use characteristics, and personality traits (i.e., conscientiousness, neuroticism).

**Conclusion:**

Overall, Californians report relatively high levels of eHealth literacy – but, those seeking to increase eHealth literacy among patients in the state may benefit from tailoring digital health information and eHealth interventions based on the significant associations found.

**Innovation:**

Few studies have examined the impacts of personality characteristics on eHealth literacy using a large sample – findings elucidate innovative pathways to enhancing eHealth literacy (e.g., accommodating online content or training interventions to those reporting low conscientiousness or high neuroticism).

## Highlights

• eHealth literacy includes skills in successfully applying online health information.
• eHealth literacy is seen as a means to empower patient decision-making.
• Data from 2020 CALSPEAKS show Californians have relatively high eHealth literacy.
• Predictors include self-rated health, religiosity, Internet use, and personality.
• Strategies promoting eHealth literacy should accommodate these identified factors.

## 1 Introduction

### 1.1 Background

Adoption and use of Internet-connected devices has steadily increased in the US over time. Per the Pew Research Center, 96 % of US adults reported using the Internet in 2024 compared to 52 % in 2000, with 79 % indicating they subscribe to broadband Internet access at home [1]. A significant proportion of Internet users access the Internet via their smartphone, with 91 % of US adults reporting use of a smartphone in 2024 compared to 35 % in 2011 [2]. Despite Internet use and adoption increasing over time, some segments of the population remain at risk for decreased access; as an example, older adults aged 65+, Black and Hispanic adults, and adults of lower socioeconomic status (i.e., lower income and lower education levels) report lower levels of home broadband use [1]. Disparities in access to Internet connectivity and applicable devices have historically been referred to as the “digital divide,” a widely used term with origins dating to the 1990s to describe gaps in information and communication technology (ICT) access [3] – but, as gaps in access have slowly closed, researchers have turned their attention to the “second-level digital divide,” defined as disparities in skills and knowledge associated with technology use [4]. Scholars of the second-level digital divide argue that while consumers and professionals may have unprecedented access to information via the Internet, some may lack the training and resources necessary to adequately find, interpret, and apply this information, contributing to a new digital-based inequality. One such emerging inequality can be found in disparities related to eHealth literacy [[5], [6], [7], [8]].

As defined by Norman and Skinner [9,10], eHealth literacy refers to an individual's ability to search for, understand, evaluate, and apply health information from digital resources. eHealth literacy is a complex concept that represents the intersection of various literacy types including traditional literacy/numeracy, computer literacy, media literacy, science literacy, information literacy, and health literacy. eHealth literacy is unique in that applicable skills require expertise in various domains. As an example, patients with strong Internet navigation skills but low health literacy may be unable to find and apply digital health information to improve their health; conversely, patients with high health literacy but low Internet experience may not be unable to find online health information necessary to make such healthcare decisions to best manage their health (e.g., finding and scheduling an appointment with an appropriate provider).

Elevated eHealth literacy can empower patients in finding and utilizing a variety of health resources to benefit their health and well-being, and it is viewed as essential for healthcare professionals in the delivery of optimal care. Having elevated levels of eHealth literacy has been shown to promote patient communication and enhance shared decision-making with providers [11,12], enhance health-focused social capital and interpersonal connections [13,14], promote patient self-efficacy [15,16], enhance health-promoting and preventative behaviors [17,18], foster disease-specific knowledge to promote self-care [19] and quality of life [20] among patients with chronic conditions, and promote collegiality among care providers and enhance shared decision-making in clinical environments [21].

Given these benefits, it is important to understand the determinants of and barriers to achieving higher levels of eHealth literacy. Digital scholars have applied theoretical frameworks such as the technology acceptance model (TAM) or the unified theory of acceptance and use of technology (UTAUT) to identify and explore these determinants and barriers, although these approaches at times have been critiqued as limiting given the decreased emphasis of sociodemographic characteristics known to correlate with digital access and inclusion [22]. van der Vaart and Dossaert's digital health literacy model, similar to Norman and Skinner's approach to eHealth literacy, underscores the importance of contextual factors (individual and societal) in measuring digital skills, including sociodemographic characteristics and one's ability to interact with the Internet [23]. Milanti et al. [24] conducted a systematic review and summarize some of the more prevalent predictors of eHealth literacy; in general, being younger, male, of higher socioeconomic status (i.e., higher education, higher income), speaking the environmental language at home (e.g., speaking English in US households), reporting higher component literacy levels (e.g., higher health literacy skills, higher information literacy skills, etc.), having higher engagement with online health information-seeking behaviors and practices, and having a more robust ICT infrastructure (e.g., increased access to ICT-related resources for troubleshooting) were associated with enhanced eHealth literacy. However, studies have sometimes exhibited contradictory findings, and the determinants and barriers to achieving elevated eHealth literacy can be context-specific. Stakeholders looking to enhance eHealth literacy among specific patient populations would best serve their constituents by assessing their specific determinants and lived barriers to achieving elevated eHealth literacy. Such assessments can elucidate how best to tailor eHealth training inventions to specific patients [25], or they may provide guidance on how to tailor online health platforms to cater to patients at risk for low eHealth literacy.

### 1.2 Study purpose

The purpose of this study was to examine factors associated with eHealth literacy and identify potential disparities in eHealth literacy in California, home to the largest and one of the most diverse adult populations in the US [26]. Findings can elucidate what segments of the California population may be at risk for lower eHealth literacy – such information can provide guidance for interventionists (particularly in California) on whom to target for eHealth training and whom to tailor training materials to, and such information can also provide guidance on digital strategies for improving health information access and use (e.g., whom to tailor California-based health websites towards, how to improve usability of California-based health websites, etc.).

## 2 Methods

### 2.1 Sample and data collection

This study used data collected from the 2020 edition of the CALSPEAKS survey, a recently disbanded project previously managed by Sacramento State University. CALSPEAKS regularly measured California public opinion on social, economic, political, and environmental issues using a representative state-wide panel survey and was notable for being the first California-focused panel using probability-based sampling methods for survey recruitment. A random sample was generated using California addresses from the US Postal Service Delivery Sequence File (stratified by region and population density), assuring that nearly all eligible state residents had a non-zero chance of being selected for inclusion in the panel. CALSPEAKS participation was restricted to non-institutionalized residents at least 18 years of age, with respondents receiving a $5 digital gift card upon completion of the survey. The 2020 survey, administered between August and September 2020, included items measuring eHealth literacy and other general technology characteristics – these items were embedded in the survey thanks to a CALSPEAKS CSU Fellowship administered through the California State University Social Science Research and Instructional Council (CSU SSRIC). Inclusion of these measures makes the CALSPEAKS survey optimal to conduct a state-specific investigation. The 2020 survey had a response rate of 60.6 % culminating in a sample of 851 respondents who completed the questionnaire (note that CALSPEAKS surveys are primarily administered online, with paper versions made available only upon request – of the 851 respondents in the 2020 survey, only 11 completed a paper-based version); restricting the analytic sample to respondents with data available on the outcome and predictor measures produced an analytic sample of 780.

### 2.2 Measures

A complete list of measures, along with descriptions and scoring, are provided in Table 1. The outcome of this investigation, eHealth literacy, was measured using a revised version of the eHEALS questionnaire originally developed by Norman and Skinner [10]. The original questionnaire was an 8-item self-assessment tool wherein respondents rated their knowledge, perceived skill, and comfort in searching for, evaluating, and successfully applying online health information to address health- and healthcare-based problems. eHEALS has been previously tested for reliability and validity [10,27], adopted in a number of US-based and international research contexts [28], and has been shown to successfully scale with actual eHealth performance measures [29]. The version used in the 2020 CALSPEAKS survey was a version produced by Sudbury-Riley et al. [27] which amended and updated the language used in the original eight items. Respondents were asked to rate how much they agree or disagree (using a 5-point Likert scale) with statements across 8 items (see Table 1 for item wording). In addition to each individual item being assigned a score from 0 to 4 (with 0 = strongly disagree/low eHealth literacy and 4 = strongly agree/high eHealth literacy), a total summed eHEALS score was generated with scores ranging from 0 to 32 (with higher scores corresponding with higher self-assessed eHealth literacy).

**Table 1:** Variable descriptions and scoring.

Variable	Description	Scoring
*GENERATION*	Recode of age into generational cohort (Generation Z, Millennials, Generation X, Baby Boomers, Silent Generation)	Dummy coded (reference = Generation Z)
*GENDER*	Gender identity (Male, Female, Genderqueer/non-binary)	Dummy-coded (reference = Male)
*NON-WHITE*	Recode of race into binary variable	0 = White, 1 = Non-white
*HISPANIC/LATINX/SPANISH*	Recode of ethnicity into binary variable	0 = non-Hispanic/LatinX/Spanish, 1 = Hispanic/LatinX/Spanish
*EDUCATION*	Highest level of education attained	0 = High school or less, 1 = some college, 2 = associate's degree, 3 = bachelor's degree, 4 = post-graduate degree
*INCOME*	Household income in past year, minimum ≤ $15,000 and maximum = $200,000+	Ranged 0–10 (higher scores = higher income levels)
*COUPLED*	Recode of marital status into binary variable	0 = single/never married/separated/divorced/widowed, 1 = married/domestic partnership/coupled and living together
*EMPLOYED*	Recode of employment status into binary variable	0 = Not employed, 1 = Employed
*BILINGUAL*	Respondent's indication of speaking multiple languages at home	0 = Speaks one language at home, 1 = Speaks two or more languages at home
*URBANICITY*	Description of living area/density (City, Suburb, Small Town, Rural Community)	Dummy-coded (reference = City)
*SELF-RATED HEALTH*	Standard measure of self-rated health (Poor, Fair, Good, Very Good, Excellent)	Ranged 0–4 (higher scores = better health)
*CHILDREN AT HOME*	Recode of number of children in household into binary variable	0 = No children present, 1 = At least 1 child present)
*PRAYS*	Recode of engagement with prayer into binary variable, proxy for religiosity	0 = Does not pray, 1 = Prays
*FREQUENCY OF USE*	Recode of frequency of Internet use (Monthly or less often, Weekly or less, Daily or less, Several times per day)	Ranged 0–3 (higher scores = higher frequency of Internet use)
*DEVICE NO.*	Number of devices respondent indicated using to access the Internet	Ranged 0–5 (higher scores = more devices)
*INTERNET ACTIVITY NO.*	Sum of activities respondent regularly performed online	Ranged 0–12 (higher scores = more activities)
*EXTRAVERSION*	Bold vs. shy, measured via two items in the BFI-10 (Likert scales)	Ranged 0–8 (higher score = more extraverted/bold)
*CONSCIENTIOUSNESS*	Organized vs. careless, measured via two items in the BFI-10 (Likert scales)	Ranged 0–8 (higher score = more conscientious/organized)
*NEUROTICISM*	Nervous vs. resilient, measured via two items in the BFI-10 (Likert scales)	Ranged 0–8 (higher score = more neurotic/nervous)
*OPENNESS*	Imaginative vs. uncreative, measured via two items in the BFI-10 (Likert scales)	Ranged 0–8 (higher score = more open/imaginative)
*AGREEABLENESS*	Cooperative vs. competitive, measured via two items in the BFI-10 (Likert scales)	Ranged 0–8 (higher score = more agreeable/cooperative)
*CA CENSUS REGION*	Recode of county residence using regions defined by CA 2020 Census (Superior California, North Coast, San Francisco Bay Area, Northern San Joaquin Valley, Central Coast, Southern San Joaquin Valley, Inland Empire, Los Angeles County, Orange County, San Diego-Imperial)	Dummy-coded (reference = San Francisco Bay Area)
*PRIMARY NEWS PREFERENCE*	Respondent selection for where they turned most often for news in the past week (Network evening news, Local TV news, Cable TV news, Non-English language TV news, Newspaper, Talk radio, Public radio or TV, Social media, Blogs or digital-only sites, Other, Does not pay attention to the news)	Dummy-coded (reference = Network evening news)
*eHEALS-1*	eHEALS item #1 (measured via Likert scale): “I know what health resources and information are available on the Internet.”	Ranged 0–4 (higher score = higher eHealth literacy)
*eHEALS-2*	eHEALS item #2 (measured via Likert scale): “I know where to find helpful health resources and information on the Internet.”	Ranged 0–4 (higher score = higher eHealth literacy)
*eHEALS-3*	eHEALS item #3 (measured via Likert scale): “I know how to find helpful health resources and information on the Internet.”	Ranged 0–4 (higher score = higher eHealth literacy)
*eHEALS-4*	eHEALS item #4 (measured via Likert scale): “I know how to use the Internet to answer my questions about health.”	Ranged 0–4 (higher score = higher eHealth literacy)
*eHEALS-5*	eHEALS item #5 (measured via Likert scale): “I know how to use the health information I find on the Internet to help me.”	Ranged 0–4 (higher score = higher eHealth literacy)
*eHEALS-6*	eHEALS item #6 (measured via Likert scale): “I have the skills I need to evaluate the health resources and information I find on the Internet.”	Ranged 0–4 (higher score = higher eHealth literacy)
*eHEALS-7*	eHEALS item #7 (measured via Likert scale): “I can tell high-quality health resources and information from low-quality health resources and information on the Internet.”	Ranged 0–4 (higher score = higher eHealth literacy)
*eHEALS-8*	eHEALS item #8 (measured via Likert scale): “I feel confident in using information from the Internet to make health decisions.”	Ranged 0–4 (higher score = higher eHealth literacy)
*eHEALS-T*	Total summed eHEALS score	Ranged 0–32 (higher score = higher eHealth literacy)

Predictor variables included a series of demographic characteristics and Internet use characteristics (detailed later in this section), census region of residence, news preferences, and personality characteristics. Census region was examined to determine if there were significant disparities in eHEALS scores based on place of residence. County residence collected in the 2020 CALSPEAKS was recoded into census regions using definitions utilized by the California 2020 Census [30] as some counties had very few respondents. Dummy variables were then created (San Francisco Bay Area = reference category).

News preferences were examined as potential predictors to determine if those reporting lower eHealth literacy would also be more likely to prefer outlets where information may be less trustworthy or less reliable, thus suggesting a novel pathway for improving eHealth literacy (e.g., targeting media literacy). The 2020 CALSPEAKS survey asked respondents where they turned most often for news in the past week. These selections were recoded into dummy variables (network evening news = reference category).

Personality characteristics (i.e., the “big 5 dimensions of personality”) were assessed using the BFI-10 adapted from Rammstedt and John [31]. The BFI-10 consists of ten items (scored using a Likert scale, ranged 0 to 4) designed to assess major personality traits including extraversion, conscientiousness, neuroticism, openness, and agreeableness. Each trait is measured by two items; for analysis, the items for each trait are combined (i.e., reverse-coded as necessary and summed) such that a total score for each trait ranges from 0 to 8 (i.e., higher scores indicating the respondent identifies more closely with that trait). Personality characteristics were selected as a possible predictor given what little work has examined its relationship with eHealth literacy – but, what work has been done has shown some significant associations. As an example, a 2024 study by Dai et al. showed that conscientiousness and openness correlated with elevated levels of eHealth literacy, whereas neuroticism and agreeableness correlated with lower levels of eHealth literacy [32].

Demographic characteristics selected for this investigation included age, gender, race, ethnicity, education, income, marital status, employment status, bilingual ability, urbanicity, self-rated health, presence of children at home, and religiosity (see Table 1 for scoring details). Internet use characteristics were also included in the analysis given the preponderance of work showing significant associations between digital abilities and eHealth literacy. Measures of Internet use included frequency of use, number of devices respondent uses to access the Internet, and breadth of Internet activities regularly performed (assessed by asking respondents to indicate what activities they regularly performed online and summing the number of activities regularly performed, scores ranged 0 to 12).

### 2.3 Analytic procedure

Ordinary least squares (OLS) regression models were run for each of the individual eHEALS items and for the summed eHEALS total to identify potential predictors of eHealth literacy. Separate models were run for census region, news preferences, and personality characteristics, partially because of what little research has examined these predictors previously but also because including these measures in a larger model could lead to an empty cell issue. A final larger model was run with demographic, Internet use characteristics, and any variable (i.e., census region, new preferences, and/or personality characteristics) previously found to be noteworthy. Regression coefficients are presented along with adjusted *R*^2^ values to assess model fit. Analyses were done using SPSS ver. 27 (IBM Corp, Armonk, NY, USA).

## 3 Results

### 3.1 Sample descriptives

Table 2 shows descriptives of the analytic sample. A majority of respondents identified as Baby Boomers (38.7 %), female (58.7 %), White (75.1 %), and non-Hispanic/LatinX/Spanish (83.7 %). Additional details on sample characteristics across predictors and outcomes can be found in Table 2, although it should be highlighted that the average score among respondents for the total summed eHEALS score was 24.62 (out of a possible 32). While there are discrepancies across studies in translating raw eHEALS scores into robust definitions of high and low eHealth literacy levels, this study applied a definition utilized in similar work [6,33,34] where scores equal to or above 18 represents high eHealth literacy (i.e., <18 = low eHealth literacy). Using this cutoff, the 2020 CALSPEAKS analytic sample demonstrated relatively high eHealth literacy levels.

**Table 2:** Analytic sample descriptive statistics (*N* = 780).

Variable	Mean (SD) or %
*GENERATION*	
Generation Z	5.1 %
Millennials	22.1 %
Generation X	24.4 %
Baby Boomers	38.7 %
Silent Generation	9.7 %
*GENDER*	
Male	40.5 %
Female	58.7 %
Genderqueer/non-binary	0.8 %
*NON-WHITE*	24.9 %
*HISPANIC/LATINX/SPANISH*	16.3 %
*EDUCATION*	2.76 (1.26)
*INCOME*	6.49 (2.61)
*COUPLED*	56.3 %
*EMPLOYED*	51.9 %
*BILINGUAL*	23.5 %
*URBANICITY*	
City	51.0 %
Suburb	28.3 %
Small town	13.2 %
Rural community	7.4 %
*SELF-RATED HEALTH*	2.58 (0.95)
*CHILDREN AT HOME*	22.7 %
*PRAYS*	72.8 %
*FREQUENCY OF USE*	2.89 (0.40)
*DEVICE NO.*	2.18 (0.83)
*INTERNET ACTIVITY NO.*	6.83 (2.61)
*EXTRAVERSION*	4.02 (2.06)
*CONSCIENTIOUSNESS*	6.28 (1.59)
*NEUROTICISM*	3.22 (2.07)
*OPENNESS*	5.33 (1.82)
*AGREEABLENESS*	5.18 (1.72)
*CA CENSUS REGION*	
Superior California	14.6 %
North Coast	4.2 %
San Francisco Bay Area (reference)	22.6 %
Northern San Joaquin Valley	3.1 %
Central Coast	8.1 %
Southern San Joaquin Valley	5.3 %
Inland Empire	7.6 %
Los Angeles County	19.5 %
Orange County	7.3 %
San Diego – Imperial	7.8 %
*PRIMARY NEWS PREFERENCE*	
Network evening news (reference)	13.2 %
Local TV news	10.8 %
Cable TV news	13.7 %
Non-English language TV news	0.6 %
Newspaper	21.2 %
Talk radio	1.5 %
Public radio or TV	10.5 %
Social media	14.6 %
Blogs or digital-only sites	2.6 %
Other	8.2 %
Does not pay attention to the news	3.1 %
*eHEALS-1*	3.02 (0.79)
*eHEALS-2*	3.06 (0.81)
*eHEALS-3*	3.19 (0.72)
*eHEALS-4*	3.28 (0.70)
*eHEALS-5*	3.15 (0.74)
*eHEALS-6*	3.19 (0.79)
*eHEALS-7*	3.03 (0.88)
*eHEALS-8*	2.69 (0.91)
*eHEALS-T*	24.62 (5.00)

*Note*. Statistics presented as means with standard deviations or as percentage of sample with that attribute.

### 3.2 Census region

Table 3 displays the mean scores for each individual eHEALS item by California census region. Table 4 shows the regression results wherein the eHEALS total summed score is regressed on California census region (note that regression results wherein individual eHEALS items are regressed onto census region are presented in Supplementary Table 1). In comparing the mean scores displayed in Table 3 and the regression results in Table 4, all regions showed higher (but mostly non-significant) average eHEALS scores compared to the reference category. Exceptions were found in the Inland Empire and the San Diego Imperial regions: restricting our view to the total summed eHEALS score (i.e., Table 4 regression results), Inland Empire respondents scored 1.893 points higher on average (*p* < 0.05) and San Diego Imperial residents scored 2.060 points higher on average (*p* < 0.01). The adjusted *R*^2^ for these models showed that census region alone accounted for very little variance in eHEALS scores, indicating the relatively weak predictive power of census region overall. Because of this (and to minimize the risk of an empty cell issue), census region was not included in the final models.

**Table 3:** Means for individual eHEALS items by CA census region (*N* = 780).

Predictor	Frequency	*eHEALS-1*	*eHEALS-2*	*eHEALS-3*	*eHEALS-4*	*eHEALS-5*	*eHEALS-6*	*eHEALS-7*	*eHEALS-8*
*CA CENSUS REGION*									
Los Angeles County	152	3.00 (0.83)	3.00 (0.90)	3.16 (0.77)	3.32 (0.79)	3.14 (0.76)	3.16 (0.81)	3.01 (0.95)	2.69 (0.89)
Superior California	114	2.97 (0.88)	3.06 (0.85)	3.18 (0.79)	3.28 (0.71)	3.17 (0.73)	3.16 (0.84)	3.01 (0.92)	2.62 (0.92)
North Coast	33	2.94 (0.93)	3.00 (0.87)	3.15 (0.67)	3.30 (0.73)	3.12 (0.74)	3.45 (0.62)	3.30 (0.81)	2.79 (0.86)
San Francisco Bay Area	176	2.97 (0.78)	3.01 (0.81)	3.13 (0.70)	3.20 (0.70)	3.07 (0.76)	3.11 (0.82)	2.92 (0.90)	2.60 (0.96)
North San Joaquin Valley	24	3.00 (0.66)	3.13 (0.74)	3.30 (0.55)	3.25 (0.61)	3.13 (0.68)	3.00 (0.83)	3.04 (0.86)	2.38 (0.77)
Central Coast	63	3.03 (0.69)	3.16 (0.65)	3.27 (0.65)	3.35 (0.54)	3.17 (0.61)	3.25 (0.69)	2.94 (0.93)	2.51 (0.90)
Southern San Joaquin Valley	41	3.05 (0.74)	2.95 (0.80)	3.07 (0.69)	3.17 (0.77)	3.05 (0.80)	3.17 (0.80)	3.10 (0.86)	2.68 (0.88)
Inland Empire	59	3.22 (0.72)	3.29 (0.67)	3.34 (0.71)	3.41 (0.62)	3.37 (0.61)	3.32 (0.78)	3.08 (0.90)	2.86 (0.92)
Orange County	57	3.05 (0.77)	2.96 (0.82)	3.05 (0.81)	3.21 (0.67)	3.05 (0.81)	3.18 (0.80)	3.09 (0.74)	2.89 (0.79)
San Diego - Imperial	61	3.23 (0.67)	3.23 (0.69)	3.41 (0.53)	3.41 (0.72)	3.26 (0.81)	3.36 (0.63)	3.21 (0.71)	2.95 (0.90)

*Note*. Mean statistics presented as Mean (SD).

**Table 4:** eHEALS sum total score regressed on CA census region (*N* = 780).

Predictor	*eHEALS-T*
*CA CENSUS REGION*^a^	
Los Angeles County	0.475
Superior California	0.450
North Coast	1.055
North San Joaquin Valley	0.203
Central Coast	0.677
Southern San Joaquin Valley	0.238
Inland Empire	1.893*
Orange County	0.486
San Diego - Imperial	2.060**
*ADJUSTED R*^*2*^	0.005

*Note*. Significant relationships shown as: *p < 0.05, **p < 0.01, ***p < 0.001.

a Reference category: San Francisco Bay Area.

### 3.3 News preferences

Table 5 shows the mean scores for each individual eHEALS item by news preferences, and Table 6 shows the regression results wherein the eHEALS total summed score is regressed on news preferences (note that regression results for the individual eHEALS items are displayed in Supplementary Table 2). Restricting our view to the regression results in Table 6, those who preferred to access the news by cable TV, newspaper, public radio or TV, and “other” scored significantly higher on the total summed eHEALS measure compared to the reference category. Interestingly, a significant difference in mean total eHEALS scores was also found when comparing the “does not pay attention” group to the comparison group, such that those who indicated they do not pay attention to the news scored 2.288 points higher in total eHealth literacy (*p* < 0.05). This is somewhat counterintuitive, as engagement with the news should likely be reflective of media literacy (a component of eHealth literacy) – it would be expected that those not paying attention to the news would score *lower* in eHealth literacy, not *higher*. These results can possibly be explained by the timing of CALSPEAKS data collection; as data were collected a few months prior to the 2020 presidential election, it is possible respondents were indicating they did not pay attention to *political* news (but did still engage with more general news items, which would still bolster media literacy and, as an extension, eHealth literacy). Of note is that the adjusted *R*^2^ for these models showed that news preferences alone accounted for very little variance in eHEALS scores – like census region, this variable was thus deemed as having weak overall predictive power and was not included in the final models.

**Table 5:** Means for individual eHEALS items by news preference (*N* = 780).

Predictor	Frequency	*eHEALS-1*	*eHEALS-2*	*eHEALS-3*	*eHEALS-4*	*eHEALS-5*	*eHEALS-6*	*eHEALS-7*	*eHEALS-8*
*PRIMARY NEWS PREFERENCE*									
Network News	103	2.92 (0.74)	2.88 (0.77)	3.08 (0.74)	3.13 (0.68)	3.04 (0.68)	3.01 (0.73)	2.77 (0.85)	2.55 (0.81)
Local TV	84	3.08 (0.76)	3.12 (0.83)	3.19 (0.75)	3.25 (0.73)	3.05 (0.82)	2.99 (0.92)	2.79 (0.96)	2.61 (0.96)
Cable TV	107	3.12 (0.69)	3.20 (0.67)	3.27 (0.58)	3.36 (0.60)	3.23 (0.68)	3.34 (0.73)	3.09 (0.91)	2.79 (0.93)
Non-English TV	5	3.00 (0.71)	2.80 (1.10)	2.80 (1.10)	3.00 (0.71)	3.00 (0.71)	2.60 (0.89)	2.80 (0.84)	2.20 (0.84)
Newspaper	165	3.05 (0.73)	3.14 (0.77)	3.22 (0.68)	3.33 (0.66)	3.21 (0.72)	3.28 (0.78)	3.13 (0.87)	2.69 (0.90)
Talk Radio	12	3.00 (1.04)	3.17 (1.11)	3.25 (0.97)	3.33 (0.89)	3.33 (0.65)	3.50 (0.67)	3.25 (0.62)	2.67 (0.98)
Public Radio or TV	82	3.10 (0.71)	3.16 (0.71)	3.30 (0.68)	3.34 (0.65)	3.21 (0.68)	3.27 (0.74)	3.17 (0.81)	2.65 (0.89)
Social Media	114	2.86 (0.97)	2.82 (0.96)	3.02 (0.88)	3.23 (0.82)	3.04 (0.83)	3.11 (0.86)	3.10 (0.93)	2.73 (0.93)
Blogs or Digital site	20	3.20 (0.52)	3.00 (0.65)	3.10 (0.55)	3.35 (0.59)	3.20 (0.62)	3.10 (0.72)	2.90 (0.72)	2.65 (0.81)
Other	64	3.09 (0.83)	3.14 (0.83)	3.33 (0.59)	3.30 (0.79)	3.19 (0.81)	3.30 (0.71)	3.06 (0.87)	2.73 (0.91)
Does not pay attention	24	3.00 (1.02)	3.17 (0.82)	3.21 (0.72)	3.42 (0.78)	3.29 (0.81)	3.38 (0.65)	3.17 (0.70)	3.04 (1.04)

*Note*. Mean statistics presented as Mean (SD).

**Table 6:** eHEALS sum total score regressed on news preference (*N* = 780).

Predictor	*eHEALS-T*
*PRIMARY NEWS PREFERENCE*^a^	
Local TV	0.693
Cable TV	2.033**
Non-English TV	−1.179
Newspaper	1.676**
Talk Radio	2.121
Public Radio or TV	1.816*
Social Media	0.516
Blogs or Digital site	1.121
Other	1.762*
Does not pay attention	2.288*
*ADJUSTED R*^*2*^	0.010

*Note*. Significant relationships shown as: *p < 0.05, **p < 0.01, ***p < 0.001.

a Reference category: Network News.

### 3.4 Personality characteristics

Regression results wherein eHEALS items and the total summed score are regressed on personality characteristics are displayed in Table 7. Openness showed no significant associations with any eHEALS measure, and both extraversion and agreeableness showed very little associations. Conscientiousness and neuroticism, in contrast, showed consistently significant associations with eHealth literacy across several eHEALS measures. Respondents who scored higher in conscientiousness reported, on average, significantly higher eHEALS scores, and respondents who scored higher in neuroticism scored, on average, significantly lower eHEALS scores. The *R*^2^ values for these models showed that personality characteristics collectively accounted for more of the variance in eHEALS scores compared to both census region and news preferences. Because of this, and due to the relatively consistent significant results of conscientiousness and neuroticism, personality characteristics were included in the final regression models.

**Table 7:** eHEALS items and sum total score regressed on personality characteristics (*N* = 780).

Predictor	*eHEALS-1*	*eHEALS-2*	*eHEALS-3*	*eHEALS-4*	*eHEALS-5*	*eHEALS-6*	*eHEALS-7*	*eHEALS-8*	*eHEALS-T*
Extraversion	0.006	−0.003	−0.003	0.001	0.013	0.002	−0.010	−0.041*	−0.034
Conscientiousness	0.062**	0.071***	0.071***	0.039*	0.056**	0.068***	0.048*	0.045*	0.460***
Neurotic	−0.029*	−0.025	−0.021	−0.036**	−0.030*	−0.048**	−0.057***	−0.070***	−0.315**
Openness	−0.008	0.001	−0.007	0.002	−0.007	−0.015	−0.008	−0.035	−0.076
Agreeableness	0.007	−0.001	0.007	0.003	−0.013	−0.021	−0.054**	−0.012	−0.084
*ADJUSTED R*^*2*^	0.023	0.021	0.027	0.019	0.023	0.035	0.027	0.033	0.038

*Note*. Significant relationships shown as: *p < 0.05, **p < 0.01, ***p < 0.001.

### 3.5 Full models

Table 8 displays the results of the final models wherein eHEALs items and the total summed score are regressed on the demographic characteristics (including Internet use characteristics) and on the personality characteristics measures. Some variables sporadically exhibited significant associations with one or a few individual eHEALS items (e.g., older generations of respondents scored lower on the eHEALS item related to differentiating between high and low quality online health resources and information, or *eHEALS-7*) but did not show a significant association with the summed total eHealth literacy measure. Variables which predicted multiple eHEALS items and the summed total measure included: self-rated health (i.e., those in better health reported higher eHealth literacy, *p* < 0.05), the proxy measure for religiosity (i.e., those who engaged in prayer scored lower eHealth literacy, *p* < 0.01), frequency of Internet use (i.e., those who used the Internet more often scored higher eHealth literacy, *p* < 0.05), breadth of activities done online (i.e., those who regularly performed more activities online scored higher eHealth literacy, *p* < 0.01), conscientiousness (i.e., those who identified as more organized and careful scored higher eHealth literacy, *p* < 0.01), and neuroticism (i.e., those who identified as more nervous and less resilient scored lower eHealth literacy, *p* < 0.01). The *R*^2^ for the final models were all below 0.1, indicating that the predictors included in each model accounted for less than 10 % of the variance in the outcome. The *R*^2^ for the final model for *eHEALS-T* was 0.078, indicating that additional variables may be incorporated which better account for the variance in eHealth literacy scores.

**Table 8:** Final OLS regression models predicting eHEALS items and sum total score (*N* = 780).

Predictor	*eHEALS-1*	*eHEALS-2*	*eHEALS-3*	*eHEALS-4*	*eHEALS-5*	*eHEALS-6*	*eHEALS-7*	*eHEALS-8*	*eHEALS-T*
*GENERATION*^a^									
Millennials	0.131	0.152	0.114	0.277*	0.126	−0.033	−0.192	0.033	0.607
Generation X	0.098	0.125	0.009	0.105	0.040	−0.080	−0.428**	−0.014	−0.145
Baby Boomers	0.245	0.448	0.217	0.239	0.204	−0.050	−0.486**	0.048	0.865
Silent Generation	0.096	0.355*	0.093	0.037	0.076	−0.236	−0.437*	−0.097	−0.111
*GENDER*^b^									
Female	0.014	0.012	0.043	0.009	−0.017	0.041	−0.350	−0.170	−0.102
Genderqueer/non-binary	0.182	0.126	0.021	0.200	−0.162	0.038	0.212	0.051	0.669
*NON-WHITE*	0.042	0.035	−0.032	−0.030	0.070	0.023	−0.-75	0.045	0.078
*HISPANIC/LATINX/SPANISH*	0.027*	0.036	−0.006	−0.089	−0.035	0.046	0.001	0.140	0.118
*EDUCATION*	0.053*	0.024	0.005	0.019	0.024	0.030	0.073**	0.020	0.248
*INCOME*	−0.014	−0.010	0.008	−0.010	0.001	−0.010	−0.009	−0.037*	−0.082
*COUPLED*	−0.043	−0.012	−0.041	−0.014	−0.031	0.070	−0.020	0.101	0.010
*EMPLOYED*	0.018	0.132	0.047	0.013	0.052	−0.048	−0.033	0.131	0.312
*BILINGUAL*	−0.061	0.019	0.009	0.056	0.011	0.038	0.084	−0.029	0.128
*URBANICITY*^c^									
Suburb	−0.012	−0.053	−0.005	0.010	−0.022	−0.076	−0.028	0.038	−0.148
Small town	−0.039	0.002	−0.019	0.015	−0.040	0.146	0.152	−0.030	0.187
Rural community	−0.116	−0.025	−0.074	−0.020	0.014	0.040	0.212	−0.114	−0.083
*SELF-RATED HEALTH*	0.055	0.044	0.043	0.061	0.049	0.084**	0.034	0.097**	0.467*
*CHILDREN AT HOME*	0.155*	0.221	0.176*	0.087	0.125	0.035	0.028	0.025	0.852
*PRAYS*	−0.105	−0.109	−0.099	−0.129	−0.083	−0.172**	−0.268***	−0.113	−1.077**
*FREQUENCY OF USE*	0.035	0.122	0.186*	0.182*	0.173*	0.201*	0.220*	0.016	1.134*
*DEVICE NO.*	−0.001	0.000	0.018	0.052	0.025	0.009	0.030	−0.015	0.118
*INTERNET ACTIVITY NO.*	0.023	0.030*	0.018	0.023*	0.040***	0.025	0.024	0.047***	0.231**
*EXTRAVERSION*	0.008	−0.005	−0.003	0.003	0.013	0.002	−0.010	−0.039*	−0.032
*CONSCIENTIOUSNESS*	0.052*	0.054**	0.056*	0.028	0.048*	0.059**	0.058**	0.046*	0.401**
*NEUROTICISM*	−0.022	−0.012	−0.016	−0.030*	−0.022	−0.049**	−0.065***	−0.061***	−0.276**
*OPENNESS*	−0.012	−0.001	−0.006	0.001	−0.004	−0.017	−0.012	−0.030	−0.080
*AGREEABLENESS*	−0.012	0.004	0.015	0.016	−0.003	−0.014	−0.035	−0.003	−0.010
*ADJUSTED R*^*2*^	0.029	0.047	0.049	0.067	0.061	0.068	0.090	0.068	0.078

*Note*. Significant relationships shown as: *p < 0.05, **p < 0.01, ***p < 0.001.

a Reference category: Generation Z.

b Reference category: Male.

c Reference category: City.

## 4 Discussion and conclusion

### 4.1 Discussion

As eHealth literacy is seen as a means of empowering patients in healthcare decision-making [35] and an essential tool for providers in delivering optimal care [36], it is important to elucidate the predictors and barriers of attaining higher eHealth literacy across different contexts. This study sought to explore the predictors of eHealth literacy in a sample of California residents. Using 2020 CALSPEAKS survey data, findings generated from OLS regression models found that the strongest and most consistent predictors of eHealth literacy (as measured by the self-assessed eHEALS survey) included self-rated health, a proxy measure for religiosity, measures of Internet use, and personality characteristics (i.e., conscientiousness, neuroticism). Other measures showed insignificant or less consistent relationships with eHealth literacy measures. To the authors' knowledge, this is the first study to profile eHealth literacy across California, which was relatively strong overall.

Most demographic characteristics were found to be weak and inconsistent predictors of eHealth literacy. This was somewhat unexpected given studies which showed the contrary, particularly with regards to education and income [24]. Previous analysis of CALSPEAKS data has shown significant relationships between education and eHealth literacy, although the sample was restricted to older adults [37]. Given the known associations between education level and health information-seeking behaviors [38], it may be that education is important *but* there are other variables (e.g., technology use-related characteristics) that are stronger drivers in predicting eHEALS scores among California residents.

Demographic characteristics which did show somewhat-repeated significant associations with eHealth literacy were self-rated health and a proxy measure for religiosity. The former was expected given previous work with similar findings [39,40]. The association between religiosity and the outcome measures was more interesting, as there appears to be a paucity of work which has examined this relationship. The results imply those who are more religious or engage in more religious activities are more likely to have lower eHealth literacy scores, at least in California. It is beyond the scope of this study to parse out the specific mechanisms driving this relationship, but it does underscore an important point: for those looking to enhance eHealth literacy among California residents, religious institutions (e.g., churches, mosques, synagogues, etc.) may be apt locations to conduct eHealth literacy training interventions.

Internet use characteristics, particularly frequency of use and number of activities typically done online, also showed repeated significant associations with individual eHEALS measures and the total summed score. Given that computer literacy represents a significant component of overall eHealth literacy per Norman and Skinner's model [9,10], Internet skill and comfortability would thus be expected to have a significant positive relationship with eHEALS measures. Previous work has shown similar results wherein Internet use characteristics predicted eHealth literacy scores [14,41,42], including previous analyses of CALSPEAKS data examining eHealth literacy among older Californians [37]. Stakeholders looking to promote eHealth literacy in California should be mindful of segments of the population where Internet adoption and acceptance is lower; in bolstering Internet skills and comfortability, these segments may also see an increase in their eHealth literacy.

### 4.2 Innovation

A notable finding from the regression analyses was the significant relationship between personality characteristics and eHealth literacy. Conscientiousness and neuroticism showed to be the most consistent predictors even when controlling for all other variables such that higher levels of conscientiousness was associated with higher levels of eHealth literacy, and higher levels of neuroticism was associated with lower levels of eHealth literacy. Few studies have explored the relationship between personality characteristics and eHealth literacy; as an example, Dai et al. [32] explored the relationship between personality characteristics, eHealth literacy, and quality of life among older adults and found similar relationships. Findings from CALSPEAKS suggest that those with lower levels of conscientiousness (i.e., are less organized and more careless) and those with higher levels of neuroticism (i.e., are more nervous and less resilient) may be at risk for lower eHealth literacy and thus may benefit from targeted intervention; these findings also imply that such interventions may be more efficacious if they are carefully organized and if they minimize anxiety during instruction.

Taken together, analyses of CALSPEAKS data showed that the most consistently significant predictors of eHealth literacy were self-rated health, religiosity, frequency of Internet use, number of activities typically done when online, conscientiousness, and neuroticism. For interventionists looking to enhance eHealth literacy levels in California, recommendations based on these findings to consider include tailoring interventions to (and targeting) those in fair or poor health, those with lower digital literacy and experience, those with lower organizational skills (i.e., lower conscientiousness), and those experiencing heightened nervousness or anxiety (i.e., higher neuroticism). As an example, an interventionist may target California older adults (i.e., at risk for poorer health and lower digital literacy/experience) and design an in-person eHealth class which addresses the basics of using the Internet and performing a general search, scaling the lessons up as the learner becomes more comfortable with their skills. Providing regular structured instruction with a tailored instructional manual can better accommodate potential older learners with lower conscientiousness; to accommodate those with higher neuroticism, stress reduction activities (e.g., using the Internet for entertainment) interspersed with formal instruction can mitigate feelings of nervousness.

In addition, interventionists may better enhance community eHealth literacy through partnerships with religious institutions. Those who engage in California-focused health information dissemination in a digital space should also be mindful of their audience and the potential for lower eHealth literacy; to this extent, digital health information can also be tailored based on the findings from this study (e.g., making California public health websites user-friendly even for those with little Internet experience and having this information meaningfully organized to accommodate those with lower levels of conscientiousness).

### 4.3 Limitations

As with all scholarship, this study is not without limitations. CALSPEAKS data is cross-sectional and thus causality cannot be proven. The analytic sample is not representative of the California adult population and thus results cannot be generalized to this group; while survey weights were available to more closely approximate the California population, their preliminary use drastically changed the results found in the regression analyses (with some being counterintuitive based on theory and previous research), and a decision was made not to include them in the analyses presented here. Similarly, the sample is likely not entirely representative of the California population given the modality of the CALSPEAKS survey (i.e., CALSPEAKS is primarily completed online and thus the sample is likely biased to more technologically-capable adults) and given that, while available in English and Spanish, the sample consisted of almost entirely English-speaking respondents (i.e., the survey does not account for the significant number of California residents for whom Spanish is the primary language spoken at home).

Finally, while the eHEALS measure is a validated and oft-used assessment of eHealth literacy [28], it is a self-assessed measure rather than a strict test of literacy which may bias the results (although studies have shown apt correlations between self-assessed and performed eHealth literacy [29]). Despite extensive use, due in part to its ease of implementation, eHEALS has been criticized for its single-factor structure (which may not adequately capture the breadth of Internet activities and associated skills) and the potential for social desirability bias (i.e., respondents rating their eHealth literacy skills highly to avoid admitting lower skills) [43,44]. eHealth literacy scholarship should continue exploring and implementing alternative measures of eHealth literacy; as an example, Petrič and Atanasova have proposed an extended version of the eHEALS scale incorporating additional constructs which has shown early promise [43,44], and van der Vaart and Drossaert's Digital Health Literacy Instrument (DHLI) also builds on eHEALS by examining multiple constructs of eHealth literacy [23] – these measures are self-assessed, however, and thus do not completely eliminate the potential for social desirability bias.

Future work exploring eHealth literacy in California and elsewhere should account for these limitations (i.e., limitations in survey design, limitations in measurement). Future work should also explore the efficacy and impacts of tailored interventions designed specifically for California residents.

### 4.4 Conclusion

To best promote eHealth literacy among patients, it is important to elucidate the factors which may promote or inhibit successful development of eHealth literacy-related knowledge and skills; these factors may be different, or operate uniquely, in different contexts (i.e., locations), and barriers to attaining higher eHealth literacy may prevent patients and providers from successfully finding and using necessary online health- and healthcare-related information. This study sought to profile the state of eHealth literacy in California and found that the strongest and most consistent predictors of eHealth literacy included self-rated health, a proxy measure for religiosity, measures of Internet use, and personality characteristics (i.e., conscientiousness, neuroticism). Efforts to promote eHealth literacy among California residents should account for these factors as they may provide guidance on how best to tailor health promotion materials (e.g., consider making health information websites easier to navigate for those with lower digital literacy), whom to target for eHealth interventions (e.g., consider those in poorer health), how to tailor eHealth training materials (e.g., create structured, organized, low-stakes classes which account for characteristics like low conscientiousness or high neuroticism), and how best to provide eHealth interventions in the community (e.g., partner with religious institutions). Through thoughtful and purposeful efforts, applicable stakeholders can enhance eHealth literacy in California and thus promote the health and well-being of patients and providers.

## CRediT authorship contribution statement

**Ronald W. Berkowsky:** Writing – review & editing, Writing – original draft, Visualization, Project administration, Methodology, Investigation, Funding acquisition, Formal analysis, Conceptualization. **Brandon Almanza:** Writing – original draft, Visualization, Investigation, Formal analysis. **Jasmine Garcia:** Writing – original draft, Visualization, Investigation, Formal analysis. **Noah Graza:** Writing – original draft, Visualization, Investigation, Formal analysis.

## Funding

The project described was supported by student awards administered as part of the 2022 Summer Undergraduate Research Fellowship (SURF) program at CSUCI.

## Declaration of competing interest

The authors have no conflicts of interest to report.

## Appendix A Supplementary data

Supplementary material Image 1
