# Supplementary material for: Factors predicting eHealth literacy: A profile of California, USA
Source: PEC Innov. 2025 Jun 28;7:100410. doi: 10.1016/j.pecinn.2025.100410 (PMC12270070; doi:10.1016/j.pecinn.2025.100410)
Supplement: Supplementary file 1 — Supplementary material [file mmc1.docx]

**APPENDIX**

| Supplementary Table 1 | | | | | | | | | |
| --- | --- | --- | --- | --- | --- | --- | --- | --- | --- |
|  | | | | | | | | | |
| *Individual eHEALS Items Regressed on Census Region (N = 780)* | | | | | | | | | |
|  | | | | | | | | | |
| Predictor | *eHEALS-1* | *eHEALS-2* | *eHEALS-3* | *eHEALS-4* | *eHEALS-5* | *eHEALS-6* | *eHEALS-7* | *eHEALS-8* |  |
|  |  |  |  |  |  |  |  |  |  |
| *CA CENSUS REGION* |  |  |  |  |  |  |  |  |  |
| Los Angeles County | 0.028 | -0.011 | 0.033 | 0.124 | 0.071 | 0.050 | 0.086 | 0.094 |  |
| Superior California | 0.002 | 0.050 | 0.059 | 0.082 | 0.093 | 0.050 | 0.088 | 0.026 |  |
| North Coast | -0.032 | -0.011 | 0.027 | 0.104 | 0.047 | 0.347* | 0.383* | 0.191 |  |
| North San Joaquin Valley  Central Coast  Southern San Joaquin Valley  Inland Empire  Orange County  San Diego Imperial | 0.028  0.060  0.077  0.249*  0.081  0.258* | 0.114  0.118  0.140  0.121*  0.123  0.120 | 0.167  0.145  -0.052  0.214*  -0.072  0.285** | 0.051  0.150  -0.028  0.208  0.012  0.211* | 0.051  0.101  -0.025  0.299*  -0.021  0.188 | -0.108  0.146  0.063  0.214  0.067  0.253* | 0.121  0.016  0.177  0.164  0.167  0.293* | -0.222  -0.089  0.086  0.268*  0.298*  0.354** |  |
| *ADJUSTED R^2^* | 0.001 | 0.003 | 0.007 | 0.002 | 0.002 | 0.004 | 0.001 | 0.012 |  |
|  |  |  |  |  |  |  |  |  |  |
| *Note*. Significant relationships shown as: **p* < 0.05, ***p* < 0.01, ****p* < 0.001.  ^a^ Reference category: San Francisco Bay Area. | | | | | | | | | |

| Supplementary Table 2 | | | | | | | | | |
| --- | --- | --- | --- | --- | --- | --- | --- | --- | --- |
|  | | | | | | | | | |
| *Individual eHEALS Items Regressed on News Preference (N = 780)* | | | | | | | | | |
|  | | | | | | | | | |
| Predictor | *eHEALS-1* | *eHEALS-2* | *eHEALS-3* | *eHEALS-4* | *eHEALS-5* | *eHEALS-6* | *eHEALS-7* | *eHEALS-8* |  |
|  |  |  |  |  |  |  |  |  |  |
| *PRIMARY NEWS PREFERENCE*^a^ |  |  |  |  |  |  |  |  |  |
| Local TV | 0.161 | 0.236* | 0.113 | 0.124 | 0.009 | -0.022 | 0.019 | 0.054 |  |
| Cable TV | 0.209 | 0.313* | 0.193 | 0.238** | 0.195 | 0.327** | 0.326** | 0.232 |  |
| Non-English TV | 0.078 | -0.083 | -0.278 | -0.126 | -0.039 | -0.410 | 0.033 | -0.353 |  |
| Newspaper  Talk Radio  Public Radio or TV  Social Media  Blogs or Digital site  Other  Does not pay attention | 0.132  0.078  0.175  -0.063  0.278  0.171  0.078 | 0.256**  0.283  0.275*  -0.059  0.117  0.257*  0.283 | 0.147  0.172  0.227*  -0.060  0.022  0.250*  0.131 | 0.207**  0.207  0.215*  0.102  0.224  0.171  0.290 | 0.167  0.294  0.168  -0.004  0.161  0.149  0.253 | 0.269**  0.490*  0.259*  0.096  0.090  0.287*  0.365* | 0.360**  0.483  0.404**  0.330**  0.133  0.296*  0.400* | 0.138  0.113  0.093  0.175  0.097  0.181  0.488* |  |
| *ADJUSTED R^2^* | 0.002 | 0.017 | 0.009 | 0.001 | 0.002 | 0.021 | 0.017 | 0.000 |  |
|  |  |  |  |  |  |  |  |  |  |
| *Note*. Significant relationships shown as: **p* < 0.05, ***p* < 0.01, ****p* < 0.001.  ^a^ Reference category: Network News. | | | | | | | | | |
